# COVIDomics: The Proteomic and Metabolomic Signatures of COVID-19

**DOI:** 10.3390/ijms23052414

**Published:** 2022-02-22

**Authors:** Michele Costanzo, Marianna Caterino, Roberta Fedele, Armando Cevenini, Mariarca Pontillo, Lucia Barra, Margherita Ruoppolo

**Affiliations:** 1 Department of Molecular Medicine and Medical Biotechnology, University of Naples Federico II, 80131 Naples, Italy; michele.costanzo@unina.it (M.C.); marianna.caterino@unina.it (M.C.); armando.cevenini@unina.it (A.C.); 2 CEINGE–Biotecnologie Avanzate s.c.ar.l., 80145 Naples, Italy; fedeler@ceinge.unina.it (R.F.); pontillo@ceinge.unina.it (M.P.); barral@ceinge.unina.it (L.B.)

**Keywords:** COVID-19, SARS-CoV-2, COVIDomics, proteomics, metabolomics, multiomics, COVID-19 signature, disease progression, data integration, pandemic

## Abstract

Omics-based technologies have been largely adopted during this unprecedented global COVID-19 pandemic, allowing the scientific community to perform research on a large scale to understand the pathobiology of the SARS-CoV-2 infection and its replication into human cells. The application of omics techniques has been addressed to every level of application, from the detection of mutations, methods of diagnosis or monitoring, drug target discovery, and vaccine generation, to the basic definition of the pathophysiological processes and the biochemical mechanisms behind the infection and spread of SARS-CoV-2. Thus, the term COVIDomics wants to include those efforts provided by omics-scale investigations with application to the current COVID-19 research. This review summarizes the diverse pieces of knowledge acquired with the application of COVIDomics techniques, with the main focus on proteomics and metabolomics studies, in order to capture a common signature in terms of proteins, metabolites, and pathways dysregulated in COVID-19 disease. Exploring the multiomics perspective and the concurrent data integration may provide new suitable therapeutic solutions to combat the COVID-19 pandemic.

## 1. Introduction

**Figure 1:**
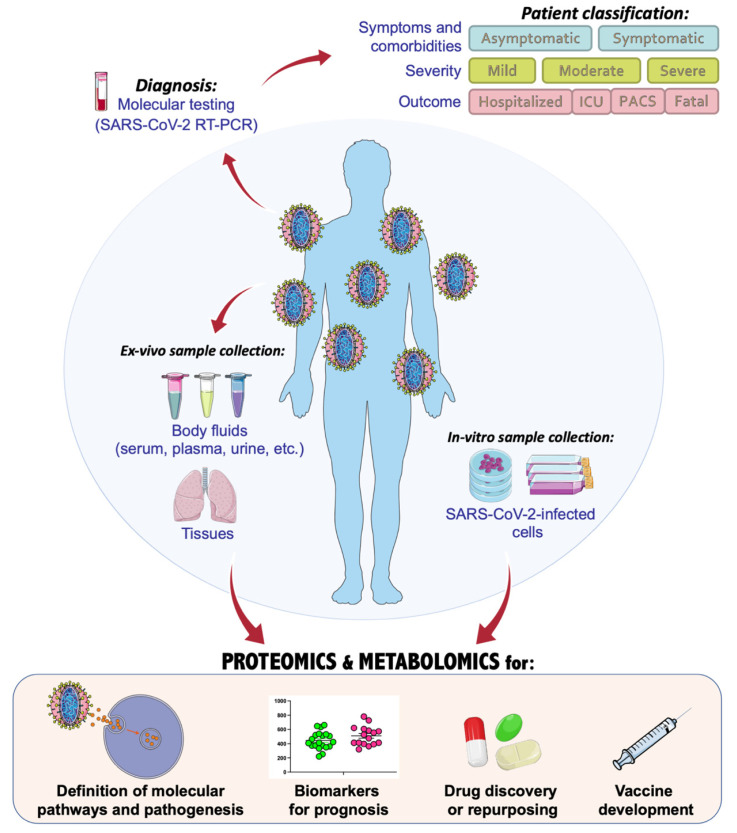
Application and integration of omics technologies to characterize the molecular biology of SARS-CoV-2 and COVID-19 pathogenic mechanisms and therapeutic approaches, with the main focus on proteomics and metabolomics investigations. This figure was drawn adapting the vector image from the Servier Medical Art bank (http://smart.servier.com/; last accessed 2 January 2022). COVID-19 = coronavirus disease 2019; SARS-CoV-2 = severe acute respiratory syndrome coronavirus 2; ICU = intensive care unit; PACS = post-acute COVID syndrome.

In the actual context of the world coronavirus disease 2019 (COVID-19) pandemic, scientists and researchers worldwide have made significant efforts to unravel the clinical and molecular aspects regarding the infection of severe acute respiratory syndrome coronavirus 2 (SARS-CoV-2) and the progression of COVID-19 disease. Meanwhile, many attempts were simultaneously made to disclose the cellular and pathophysiological processes affected in COVID-19 patients; the urgent need for pharmacological treatments prompted a rapid approach to drug repurposing and vaccines’ development, to limit the number of deaths and the spread of the infection [1,2,3,4,5]. Classification of COVID-19 disease can be made according to the clinical characteristics of patients (Figure 1). According to the high variability and heterogeneity of symptoms and comorbidities [6], patients can be categorized as symptomatic or asymptomatic, and a crescent grade of severity is usually referred to as mild, moderate, or severe. The outcome of COVID-19 patients defines their status, classifying them as hospitalized, needing intensive care unit (ICU), or fatal. Very often, even after recovery, many patients can experience post-acute COVID syndrome (PACS), during which symptoms may last for several months [7]. The World Health Organization (WHO) has organized a system score for clinical improvement and management of COVID-19 disease, based on an ordinal scale to categorize patients according to their clinical manifestations [8].

In the fight against the COVID-19 pandemic, single- and multiomics-based strategies have been largely adopted with the main aim of dissecting a plethora of aspects related to the SARS-CoV-2 infection. In fact, omics-based technologies, such as genomics, transcriptomics, proteomics, and metabolomics, can serve at every stage of COVID-19 investigations, from diagnosis and progression of the disease, to the discovery of altered molecular pathways or potential drug targets and vaccines (Figure 1).

Genomics and transcriptomics have suitably allowed to promptly identify in diverse biological matrices the mutations of SARS-CoV-2, including those responsible for the occurrence of new variants, and the changes in the coronavirus genes expression [9,10,11,12]. On the other hand, the value of proteomics and metabolomics concerns the possibility of understanding the functional alterations that depend on virus infection and its interaction with the host cell. In fact, with the increasing advance and the high throughput provided by liquid chromatography—tandem mass spectrometry (LC-MS/MS) technologies in biomedicine, the identification and quantification of several thousands of proteins and metabolites is possible in a targeted or untargeted fashion, or using the newest data-independent acquisition (DIA)-MS methods [13,14,15,16], besides the traditional data-dependent acquisition (DDA) ones [17,18,19,20,21]. With such sophisticated techniques, many aspects of the pathogenesis of COVID-19 can be disclosed, acquiring an important piece of knowledge to understand the molecular processes perturbed by virus infection and to predict or ameliorate the clinical outcome of patients by finding promising druggable targets. On this matter, even out of the scope of this review, an interactomics study performed in human cells using affinity-purification mass spectrometry found several interacting partners in 26 out of the 29 proteins of SARS-CoV-2, concluding that some of these interactors were druggable proteins [22].

In parallel, the introduction of a metabolomic workflow in a study provides a rich source of information, allowing the phenotypic biochemical and metabolic characterization of cells, tissues, or body fluids, and discovering and monitoring biomarkers or disease signatures [23,24,25]. What is more, the challenging advances in mass spectrometry and metabolomics research provided scientists with a powerful tool able to technically separate the analysis of small molecules from lipid molecules, diving into the deep of the metabolome and the lipidome, respectively [26,27]. Lipids represent the structural building blocks of cell and virus membranes, thus playing an essential role for the virus life cycle, including viral invasion and replication. Since viruses are able to modulate lipid synthesis and signaling into the host cell to produce lipids for their envelopes by creating a lipid micro-environment ideal for replication, the lipid dysregulation is suggested as a drug target to prevent coronavirus infection [28]. Furthermore, considering the high heterogeneity of the clinical manifestations of COVID-19, thus suggesting the involvement of diverse pathophysiological processes [29], multiomics approaches and data integration analyses may result as a winning choice, contributing to a better understanding of the molecular biology of SARS-CoV-2 at every level of complexity [30].

To unify all the efforts made by scientists at the omics-scale level in the research to combat the current pandemic and provide a comprehensive overview of the omics studies made in favor of COVID-19 research, we used the term COVIDomics. Hence, in this review we summarized the COVIDomics discoveries mainly based on proteomics and metabolomics studies on blood samples from patients. Many overlapping results were found in several publications from different authors for both proteomics and metabolomics applications. The relevant findings of such investigations were highlighted in this review and, finally, summarized and analyzed with bioinformatics tools in order to capture a common signature in terms of proteins, metabolites, and pathways dysregulated in COVID-19 disease.

## 2. Proteomics of COVID-19

Several proteomics investigations were conducted on different biological matrices obtained from COVID-19 patients, even though a majority of the studies published in the literature have been performed on plasma and serum. For this reason, as follows, the main findings retrieved from the reviewed publications will be predominantly grouped according to their application to blood, and only a few cases to cells. The main perspective behind the proteomics studies performed on COVID-19 was to define specific signatures across the different stages of disease severity, for the discovery of biomarkers or potential therapeutic targets. Especially for the studies based on patient-derived biofluids, the statistical and differential analyses largely differ amongst the authors. This was due to the different stages of COVID-19 disease used to perform comparisons, which were usually marked as mild, moderate, severe, critical, or divided as severe and non-severe, or hospitalized and non-hospitalized. Most of the authors used to classify patients according to the disease severity referring to the WHO ordinal scale set for clinical improvement and management of COVID-19 [8]. Table 1 groups the papers reviewed in the following sections with regard to proteomics technology and samples adopted by each author, including a summary of the main biological findings obtained from COVID-19 patient samples. 

### 2.1. Plasma Proteomics Studies

Blood proteomics and metabolomics have a great potential in the context of a global pandemic, since many samples could be obtained from patients and donors. Blood is a readily accessible biofluid and can be used as liquid biopsy to unravel systemic mechanisms of disease. Since plasma or serum reflect systemic changes, investigating the circulatory proteome and metabolome by COVIDomics can provide significant clinical and biological information [31,32]. Generally, the blood proteomics studies here reviewed were performed using MS- or non-MS-based strategies, but they commonly found association of COVID-19 with a selective inflammatory milieu.

With regards to non-MS techniques, many authors have made use of the Proximity Extension Assay (PEA) technology, which corresponds to a protein microarray particularly suitable for liquid samples that through an immunoassay provides the high throughput detection of protein biomarkers [33]. Using this technology, Patel et al. [34] performed a plasma proteome profiling of 59 COVID-19 (26 mild, 9 severe, 24 critical) patients and 28 healthy controls. The comparison “control vs. case” recognized 269 differentially expressed proteins, of which the down-regulation of neurofibromin 2 (NF2) was proposed as a specific plasma biomarker for COVID-19. The analysis of the 269 differential proteins enriched the “Cytokine-cytokine receptor interaction” as the most significant biological pathway. In addition, the abundance of six proteins (IL-6, CKAP4, Gal-9, IL-1ra, LILRB4, PD-L1) increased as the symptoms of patients increased (in the direction: control → mild → severe → critical) and, thus, was associated with COVID-19 severity. In the longitudinal study of Haljasmägi and colleagues [35], the same PEA technology was used to profile the plasma of 40 COVID-19 patients, of which 25 moderate (not requiring intensive care unit, non-ICU) and 15 severe (ICU), compared to age-matched negative controls. Mostly, markers of inflammation such as IL-6, IL-10, CXCL10 (also known as IP10), CXCL11, CCL2, CCL7, CCL8, PD-L1, and IL-18R1 were increased at the early stage of COVID-19 in ICU patients compared to non-ICU ones. Moreover, the IFNγ, an activator of CXCL9, CXCL10, and CXCL11, showed this trend. With the exclusion of IL-6, which was maintained up-regulated in several ICU patients, all these markers declined over the time. Other plasma markers with similar trends were TGFB1, Oncostatin M, S100A12, IL-7, CCL23, VEGFA, and CSF-1. These results were further validated in ICU, non-ICU and additional mild-disease patients showing increase in the triad IL-6, IL-10, CXCL10, as associated with disease severity. According to these authors, COVID-19 is associated with an inflammatory environment and strictly connected to apoptosis dysregulation, since increased level of apoptosis markers, namely CASP8, TNFSF14, TGFB1, and HGF were found compared to controls. Finally, SCARB2 and EDA2R proteins correlated with neuronal injury markers, advising above all for the involvement of neuronal impairment in COVID-19. Moreover, Zhong et al. [36] in their study identified with the PEA technology increased levels of SCARB2 in 50 mild and moderate COVID-19 patients, comparing the protein levels in the same individuals after 14 days. A total of 239 proteins was found altered and the top-50 significant ones were classified as cytokine- or immune-related. In accordance with Haljasmägi, other up-regulated proteins related to cytokine response or immune function during COVID-19 infection were CXCL10, CCL8, CLEC6A, IFNL1, IL-15, IL-18BP, IL-18R1, LAG3, LAMP3, LGALS9, SIGLEC1, SIGLEC5, and TNF. Among the differential proteins, only two were decreased, namely CCL24 and TNFRSF10C. Finally, when these data were compared with the plasma proteome response in severe-symptoms patients [37], the abundance pattern of many proteins was maintained with the exception of SCARB2, which was the most significant protein in Zhong’s study and did not show increased levels in the severe cohort.

Similar inflammatory markers were also detected by Bauer et al. [38], analyzing with a PEA array the plasma proteome of 44 non-hospitalized and 53 hospitalized COVID-19 patients, and 44 non-COVID subjects. In fact, from the group comparison of COVID-19 vs. non-COVID-19, the authors identified 14 specific inflammatory proteins that can be related to SARS-CoV-2 infection, namely CXCL5, CXCL10, CXCL11, Gal-9, IL-18, IL-18R1, IFNγ, LIF-R, MCP-2, MCP-3, MERTK, MMP-1, PD-L1, and TNF. From the comparison of hospitalized vs. non-hospitalized patients, 12 proteins including ADM, CTSL1, HGF, IL-6, IL-27, KIM1, MERTK, MMP-1, MMP-12, OPG, TNFRSF10A, and TRAIL-R2 were increased in the hospitalized group. Furthermore, these authors identified ADM, IL-6, MCP-3, TRAIL-R2, and PD-L1 as predictive for death.

With regards to MS-based approaches, Shu et al. [39] analyzed the plasma proteome collected at different times per patient of 5 fatal, 7 severe, and 10 mild COVID-19 subjects compared to 8 healthy controls via tandem mass tag (TMT) labeling and LC-MS/MS. The proteomic analysis of fatal patients compared to healthy controls detected 195 differential proteins with a grade of differential expression that reduced as severity of symptoms decreased in the mild group. This suggested that the alterations of the proteome are significantly correlated to the severity of the disease. Pathway enrichment analysis of these proteins revealed alteration of processes related to inflammation, immune cell migration and degranulation, complement system, coagulation cascades, and energy metabolism. Using a machine-learning model, 11 biomarkers were found to have the power to classify and predict the clinical outcome of COVID-19. These were further validated by authors using proteomics and ELISA assay employing the plasma derived from two additional cohorts of COVID-19 patients. The markers ORM1, ORM2, S100A9, CRP, AZGP1, CFI, SERPINA3/ACT, and LCP1/LPL were significantly increased in more severe COVID-19 conditions, whereas the levels of FETUB, CETP, and PI16 were significantly decreased.

Park et al. [40] analyzed the plasma proteome of a small cohort of three non-severe (mild) and five severe cases of COVID-19 patients. Using the BoxCar acquisition method within a high-resolution LC-MS/MS system, without performing high-abundant protein depletion they were able to identify 1639 proteins, of which 91 were differentially abundant between the mild and severe conditions. The most significant biological functions associated with this dataset were related to neutrophils activation and blood coagulation. For these categories, ALDOC, HSPA8, IQGAP2, PIGR, SERPINA1, SERPINA3, SERPINB10, and VAPA proteins were up-regulated. As conflicting results, other similar neutrophil-related categories included the down-regulated proteins BST1, CTSD, DEFA1, FTH1, PGAM1, and SIRPB1.

Moreover, Messner et al. [41] analyzed the plasma proteome of severe and mild patients of two independent COVID-19 cohorts, with a DIA-MS method. Initially, 31 (mild and severe) patients were included in the exploratory cohort to identify biomarkers, and 27 proteins were then validated in the validation cohort, constituted by 15 healthy subjects and 17 (8 mild and 9 severe) COVID-19 patients. These potential biomarkers were identified as either more or less abundant with a differential trend correlating with the disease severity, including the following proteins (or protein groups): A1BG, ACTB/ACTG1, ALB, APOA1, APOC1, C1R, C1S, C8A, CD14, CFB, CFH, CFI, CRP, FGA, FGB, FGG, GSN, HP, ITIH3, ITIH4, LBP, LGALS3BP, LRG1, SAA1, SAA1/SAA2, SERPINA10, and TF. Confirming previous results, the protein dataset enriched cellular pathways involving the complement factors, the coagulation and the inflammation cascades.

Finally, a DIA-MS study was performed by Demichev et al. [42] to characterize the time-dependent evolution of COVID-19 disease in 139 patients (classified with a variable grade). Analyzing the plasma proteomes at 687 sampling points, the authors describe a primary spike of the inflammation and systemic response, and then a gradual decrease in such response, suggestive of ongoing processes of immunomodulation, tissue repair, and metabolic recovery. Thus, 113 proteins changed according to the disease severity as markers of progression, including immune and inflammatory mediators (A2M, B2M, CD44, PIGR), complement factors (CFD, CFH, CFHR1, CFHR2) and apolipoproteins (APOA2, APOC3, APOD, APOE, APOL1). In addition, they found significant positive and negative correlation of many proteins with IL-6 amount. These proteins include acute phase proteins (APOA2, APOE, AHSG, CD14, CRP, GSN, ITIH3, ITIH4, LYZ, SAA1, SAA2, SERPINA1, SERPINA3), coagulation factors and related (FGA, FGB, FGG, F2, F12, KLKB1, PLG, SERPINC1), and complement factors (C1R, C1S, C8A, C9, CFB, CFD, CFHR5).

### 2.2. Serum Proteomics Studies

The serum proteome was analyzed by Hou et al. [43] using an in-house antibody microarray in a cohort of 15 early-stage COVID-19 patients compared to 13 influenza patients showing similar symptoms. In this analysis, 88 up-regulated proteins (including APOC3, CCL2, CCL17, CXCL8, CXCL10, FAS, IL-6, IL-21, IL-32, LEP, PRL, TNF, VEGFA, VIM) and 37 down-regulated proteins (including APOL1, APOL6, C1R, C7, C8A, CFB, CFH, CFHR5, PLG, VTN) were characterized as distinctive of SARS-CoV-2 infection, reflecting a prevalent inflammation and immune response in early COVID-19. In fact, four classes of biological processes were enriched, namely immune cell activation and migration, cellular activities, protein signaling that regulate viral infection, and blood functional systems.

Chen Y. et al. [44] profiled via DIA-MS the serum proteome of 10 moderate and 6 severe COVID-19 patients (and 10 healthy controls) during the disease and at recovery stages. A first analysis was performed to explain the long-term disturbances affecting subjects also after recovery. The proteins altered during the infection and maintained altered also after recovery enriched functions related to cholesterol transport and metabolism, including APOL1, APOA2, and SERPINA12 for moderate patients, and APOA5, APOL1, APOA2, and CSE1 for severe ones. With the same principle, a long-term disruption in the processes related to myocardium function was highlighted for both moderate and severe patients, including proteins of sarcomere function (TTN, MYH7, NEXN) or cell migration and adhesion (COL14A1, COL2A1, ITGA7, LGALS3, LOX, MMP, THBS4). A second analysis comparing severe vs. moderate patients confirmed a further impairment of processes related to cholesterol metabolism, blood coagulation, and the cardiovascular system that correlates with the disease severity. These groups include proteins such as APOA5, CES1, CFL1, HSP90AB1, ITGA7, and NEXN, which were differentially abundant in severe patients both in the disease and recovery stages.

Accordingly, the serum DIA-MS study of Kimura et al. [45] of 10 severe COVID-19 patients (5 adverse and 5 favorable prognosis) confirmed the dysregulation of proteins involved in cardiovascular injury and myocardial function in addition to alteration of the inflammatory response. Globally, 27 proteins were associated with adverse prognosis, of which 16 up-regulated (ATP6AP1, CD93, CHI3L1, CST3, CTSB, GIPC2, GRN, IGFBP6, LCN2, MB, MPO, SECTM1, SPP1, VIM, VSIG4, VWF) and 11 down-regulated (APOC1, APOC2, APOC3, APOC4, APOM, ARFIP1, IGFALS, IGFBP3, PRG4, SAA4, TTR).

The comparison of severe (*n* = 12) and non-severe (*n* = 13) COVID-19 sera patients was also profiled by DIA-MS by Lee et al. [46], revealing 46 differential proteins that identify altered GO biological processes in severe patients, as follows: IGHA2, IGLC2, and IGLV3-19 (humoral immune response); BNC2 (IFN signaling); ALB, CRP, ITIH4, LBP, RBP4, SAA1, SAA2, SERPINA3, TF, and TTR (acute phase response); AGT, HSP90AA1, and TKT (inflammatory response); APOA1, APOA2, APOA4, APOC1, APOM, and PON1 (lipid metabolism); A2M, AHSG, HRG, KNG1, LEFTY2, PF4, SEPP1, and SERPINA4 (platelet degranulation); F9, F10, SERPINA1, and SERPING1 (coagulation cascade); and ALDOA, Gc-globulin (GC), HGFAC, ITIH2, IGFALS, L1TD1, MAN1A1, MAN1C1, PI16, PIK3C2β, and PRG4.

Finally, serum proteomics was performed by D’Alessandro and colleagues [47] on 49 subjects, including 16 controls and 33 COVID-19 patients, whose severity status was inferred measuring IL-6 levels. LC-MS/MS and pathway analyses defined the IL-6 signaling as the most up-regulated pathway revealing the increase in CRP, LRG1, S100A12, SAA1, SERPINA3, SFTPB, TIMP1, and the decrease in ASH1L, CETP, CRISP3, F13B, GSN, IGFALS, IGFPB3, PARP9, PSRC1, and STOM in COVID-19 patients vs. controls. Patients’ stratification according to IL-6 levels revealed dysregulation of coagulation factors (F5, F7, F10: up-regulated; F13B, GSN: down-regulated), increase in pro-coagulant (KNG1, FGA) and anti-coagulant (PROS1) factors, coagulation/fibrinolytic cascade components (SERPINA1, SERPINA3, SERPINC1, SERPIND1, SERPINF2, CPB2), complement cascade components (C5, CFH, CFI), and antimicrobial enzymes (CST3, DEFA1, FCN2, LRG1, LYZC, ORM1), as well as several immunoglobulins.

### 2.3. Infected-Cells Proteomics Studies

Other authors have performed proteomics analyses on human cells infected by SARS-CoV-2. One example is the paper by Bojkova et al. [48], in which the proteome of Caco-2 cells was investigated using a LC-MS/MS-based SILAC (Stable Isotope Labelling by Amino acids in Cell culture) approach. At 24 h post-infection, an extensive modulation of the host-cell proteome was observed. The cluster of down-regulated proteins enriched the cholesterol metabolism, while the up-regulated proteins were components of the spliceosome and carbon metabolism (referred to as glycolysis). Using inhibitors of spliceosome and glycolysis, the authors proved that viral replication was prevented, suggesting these as potential therapeutic targets. Accordingly, the proteomic study from Grenga et al. [49] corroborated the above-mentioned findings, detecting in Vero cells an increase in RNA modifiers, such as spliceosome components, proteins of carbon metabolism, and additional proteins involved in vacuole formation and viral budding. Interestingly, the proteomics data produced by Bojkova et al. were re-analyzed by Bock [50] to get further insight into the host-cell response after SARS-CoV-2 infection. Thus, the authors highlighted dysregulation of proteins connected with the inflammatory response and chromosome segregation during mitosis.

## 3. Metabolomics of COVID-19

As for the proteomics studies, the majority of the metabolomics investigations were also performed on biofluids such as plasma or serum of COVID-19 patients at different stages to find prognostic marker metabolites, predict the evolution of the disease, and dissect the metabolic perturbations caused by SARS-CoV-2 infection. Despite gas chromatography-mass spectrometry (GC-MS) and LC-MS/MS techniques being the most frequently adopted, few studies also employed nuclear magnetic resonance (NMR) for metabolite detection. Finally, we will review the studies with focus on both the metabolome and the lipidome of COVID-19 patients, with summary of the relative main findings reported in Table 2. 

### 3.1. Plasma Metabolomics Studies

Several studies based on the metabolome profiling have shown a clear impairment of amino acid metabolism that comprises alteration of canonical amino acids and their derivatives. Danlos et al. [51] uncovered COVID-19 stage-dependent shifts in the plasma metabolome of 23 mild, 21 moderate, and 28 critical patients compared to 29 controls by both LC-MS/MS and GC-MS. The levels of 77 metabolites that include amino acids, lipids, polyamines, and sugars, as well as their derivatives, were found changed in critical patients. Precisely, arginine, aspartic acid, glutamic acid, phenylalanine, tyrosine, leucylproline, and S-adenosylmethionine were increased. Alteration in the tryptophan metabolism was found, as tryptophan can be converted into NAD (nicotinamide adenine dinucleotide) via the kynurenine pathway. In fact, tryptophan was decreased and kynurenine increased in critical patients. Furthermore, the increase in the kynurenine metabolite, namely anthranilic acid, showed a poor prognostic value. In accordance are the results of Blasco et al. [52], which showed alteration of tryptophan, nicotinate and nicotinamide (NAM), and pyrimidine metabolism. Two metabolites, namely cytosine and indole-3-acetic acid, mainly discriminated between COVID-19-positive and -negative patients, beside the other metabolites (2-aminophenol, 1-NH_2_-cyclopropane-1-carboxylate, asparagine, isoleucine, leucine, nicotinamide, uric acid and xanthine). Cytosine was described as regulator of cell metabolism in SARS-CoV-2 [53]. The indole-3-acetic acid is a breakdown product of tryptophan metabolism. The nicotinamide is a derivative of nicotinic acid serving as precursor of the coenzymes NAD+ and NADP+, indispensable for many biochemical reactions. In accordance, Heer et al. [54] found that coronavirus infection may dysregulate the NAD metabolome. 

Another study by Fraser et al. [55] confirmed an increment of kynurenine in critically-ill (ICU) COVID-19 patients, and reduced levels of arginine, sarcosine, and lysophosphatidylcholines (lysoPC). Moreover, creatinine or the creatinine/arginine ratio were found to predict ICU mortality with a 100% accuracy. LysoPC, PC, and serine were also found decreased by Sindelar et al. [56], which analyzed by untargeted LC-MS/MS metabolomics the plasma samples of 272 patients longitudinally collected at six time points, to find 22 prognostic markers that predict COVID-19 severity by changing early during the disease and being restored in the recovery phase. Among the 22 metabolites, kynurenic acid, 1-methyladenosine, phosphatidylethanolamines (PE), and ceramides (Cer) were found increased. Instead, Bizkarguenaga et al. [57] found by NMR that recovered COVID-19 patients between 3 and 10 months after diagnosis showed increased plasma levels of cholesterol, triglycerides (TG), and phospholipids. Finally, Khodadoust [58], through lipidomic analysis of 52 plasma samples, detected lipids belonging to the ceramide class increased by a 400-fold in infected patients.

### 3.2. Serum Metabolomics Studies

Besides the study of Raines [59], which also confirmed in the urine metabolome from hospitalized COVID-19 patients the impairment of energy metabolism, purine metabolism, and NAD+ synthesis, the serum metabolome of COVID-19 patients showed many similarities as compared to the plasma. In particular, the majority of the changes observed as function of the disease severity are linked to amino acid and energetic metabolism.

The untargeted metabolomic analysis of 120 COVID-19 patients by Roberts et al. [60] found 20 molecules predictive of severity or outcome, comprising an increase in deoxycytidine, ureidopropionate, and pseudouridine, as well as a decrease in uridine. Metabolites of the tryptophan/kynurenine pathway were kynurenine and kynurenic acid (increased) as well as serotonin and melatonin (decreased). Multiple short-chain acylcarnitines were found elevated in both severe and poor-outcome patients, suggesting strong impairment of energy metabolism.

Xiao et al. [61] correlated the alteration of arginine metabolism (arginine, aspartic acid, citrulline, glutamine, proline, urea), purine metabolism (adenine, adenosine, GMP, guanine, guanosine, xanthine, xanthosine), and tryptophan/NAD+ metabolism (kynurenine, nicotinamide mononucleotide (NMN), nicotinic acid) with the cytokine release syndrome and the inflammatory response (positive or negative correlation with cytokines levels) induced by SARS-CoV-2. In accordance was also the study of Li et al. [62], which showed mainly up-regulation of several metabolites such as carbohydrates and conjugates, and a clear signature that include amino acids and analogues, suggesting dysregulation of carbohydrate metabolism and amino acid metabolism as possibly connected with the disease severity in COVID-19 patients. In fact, significantly enriched pathways were: alanine, aspartate, and glutamate metabolism; arginine and proline metabolism; phenylalanine metabolism; and glycine, serine, and threonine metabolism. Of interest is also the up-regulation of five urea cycle-related metabolites (3-amino-2-piperidone, asparagine, aspartic acid, N-acetylornithine, ornithine) that highly correlated with the cytokine storm and coagulation indices in severe patients. Two works performed by GC-MS confirmed serum alteration of energy metabolism (Krebs cycle, Warburg effect) and amino acid catabolism (branched-chain amino acids, threonine, glutamine and glutamic acid) pathways (Páez-Franco et al.) [63], and also demonstrated that the combination of 2-hydroxy-3-methylbutyric acid, 3-hydroxybutyric acid, cholesterol, oleic acid, ornithine, palmitelaidic acid, and succinic acid was predictive of disease severity (Shi et al.) [64]. 

Ansone et al. analyzed the serum samples of patients at the acute phase of the disease to track the metabolites that changed during the recovery phase, finding alteration of several metabolites involved in: D-glutamine and D-glutamate metabolism (glutamine, glutamic acid), phenylalanine metabolism, and arginine and proline metabolism especially via the urea cycle (citrulline and ornithine). The decrease in tryptophan and the up-regulation of kynurenine and 3-hydroxy-DL-kynurenine in the acute phase confirm the alteration of the tryptophan/kynurenine pathway [65]. The dysregulation of glucose and energy metabolism in the COVID-19 disease progression, in particular related to the urea cycle and the tricarboxylic acid (TCA) cycle, was also suggested by Jia [66]. In fact, aspartate metabolism, urea cycle, arginine and proline metabolism, glycine and serine metabolism, and the TCA cycle were the most impacted pathways according to the alteration of 2-oxoglutaric acid, arginine, aspartic acid, citrulline, creatine, glutamine, malate, ornithine, and pyruvic acid. 

Once again, the tryptophan/kynurenine pathway was top-altered, as found by Thomas et al. [67] through reduction of tryptophan, serotonin, and indolepyruvate; as well as elevation of kynurenine, kynurenic acid, picolinic acid, and nicotinic acid; but not of anthranilic acid levels; and in combination with alterations in carbon and nitrogen metabolism. The same results were uncovered by Caterino et al. [68], which disclosed the metabolic alterations of a cohort of mild, moderate, and severe COVID-19 patients. A clear signature was determined with increased levels of lactic acid in all the disease stages, while additional dysregulations possibly affecting carbon and nitrogen metabolism were found in moderate and severe patients, including the metabolites: arachidonic acid, aspartic acid, beta-alanine, choline, glutamic acid, ornithine, phenylalanine, succinic acid, and xanthine. Enriched pathways were: glycolysis and pyruvate metabolism; D-glutamine and D-glutamate metabolism; nitrogen metabolism; pyrimidine metabolism; purine metabolism; and phenylalanine, tyrosine and tryptophan biosynthesis. The same authors analyzed the lipidome of such patients, identifying altered levels of TG and increase in Cer [69]. Dei Cas et al. [70] detected alterations of PC and glycosphingolipids, with an increase in acylcarnitines and phosphatidylethanolamines (PE), and a decrease in cholesteryl esters, diacylglycerols (DAG), lysoPE, and sphingomyelins (SM). A decrease in SM was also found by Torretta et al. [71] in severe patients, with elevation of ceramides, dihydroceramides, sphingosine, and dihydrosphingosine levels. Finally, the results by Kaur et al. showed an increase in SM, PC, and arachidonic acid, as well as elevated concentrations of tryptophan and its metabolites in the serum of severe compared to recovered COVID-19 patients [72].

## 4. Multiomics Studies of COVID-19

As previously mentioned in this review, multiomics-based technologies have been extensively employed yet to define the molecular pathways concerning SARS-CoV-2 infection and COVID-19 pathogenesis, through the discovery of disease signatures involving elevation or decrease in specific protein or metabolite markers. More generally, many variations of this disease were captured by integrative proteomics and metabolomics studies performed on blood samples from patients at different stages, despite others combine proteomics with genomics and/or transcriptomics approaches. We have included the main characteristics relative to the proteomics and metabolomics, retrieved from the multiomics studies commented below into the Table 3.

Starting from the multiomic analysis of plasma samples, Su et al. [73] identified a major shift between mild and moderate disease, and captured a specific omic signature at this point of shift. The proteomic and metabolomic analyses defined a patients’ profile dependent on disease severity, confirming previous studies. In particular, they found an increase in pro-inflammatory cytokines (e.g., CCL7, CXCL6, CXCL10, IL-6, IL-10) and immune cell activation proteins (e.g., CD244 and CD40), in combination with altered amino acid, nucleotide, and carbohydrate metabolism. Additionally, a preferential down-regulation of lipid molecules was observed toward the transition from mild to severe.

Chen Y.-M. et al. [74] analyzed the plasma of 16 severe and 50 mild COVID-19 patients by DIA-MS proteomics and NMR metabolomics. The main findings were down-regulation of the TCA cycle and glycolytic pathways and activation of the HIF-1, IL-6, TNF, T-cell receptor, and platelet signaling pathways. In detail, weighted gene co-expression network analysis (WGCNA) identified some protein modules containing the down-regulated AHSG, C4BPB, ECM1, F12, F13A1, F13B, HRG, and PROS1 that were strongly associated with activated partial thromboplastin time (APTT). By contrast, IL-6 and IL-10 were higher in severe than mild patients, and positively correlated with the increased expression of AGA, DEGS1, DSN1, LCN2, NFKB1, and PSEN1. The metabolic alterations were imputed to some lipoprotein subclasses and increased levels of TG and free cholesterol in both mild and severe patients as well as reduction of total cholesterol and phospholipids.

Krishnan et al. [75] used proteomics and metabolomics to characterize the plasma of COVID-19 patients showing significantly raised levels of cytokines and chemokines, such as IL-6. Immune-related pathways, such as cytokine-cytokine receptor interaction, chemokine signaling, TNF signaling pathway, etc., were the top enriched, including 11 proteins altered between mild and severe patients (ANGPT2, CCL23, CXCL12, CXCL13, FASLG, HGF, IL-12, MCP-3, PTN, TWEAK, VEGFA). The majority of the differential metabolites were lipids followed by amino acids. Alanine, arginine, glutamine, glycine, histidine, proline, and tryptophan were lower in COVID-19 patients; aspartic acid, glutamic acid, and phenylalanine were found at higher levels. In addition, glycolysis (3-phosphoglyceric acid, glucose, pyruvic acid, lactic acid) and TCA cycle (aconitic acid, alpha-ketoglutaric acid, citric acid) metabolites showed alteration. Plasma glucose and mannose were classified as biomarker for disease severity, despite in vitro assays showed that only high concentrations of glucose but not mannose affected viral *E-gene* expression and replication.

A proteomic and metabolomic signature during the transition of COVID-19 severity was also established by Suvarna et al. [76]. In particular, 5 metabolites (arginine, creatine, 3-hydroxyoctanoyl carnitine, 3,5-tetradecadiencarnitine, and 1-stearoyl-2-hydroxy-sn-glycero-3-PE) distinguished between severe and non-severe COVID-19, while 10 proteins resulted differential (HSPA8, IGFALS, LGALS3BP, PDIA3, PDLIM1, PFN1, PTGDS, RAP1B, SERPINA10, TGFB1). Some of these proteins are increased in severe patients, playing an important role in platelet function and thrombosis. In fact, the integrated pathway analysis enriched the complement and coagulation cascades, platelet activation and degranulation, myeloid leukocyte activation, and arginine amino acid metabolism in the severe patients.

Wu et al. [77] performed a trans-omics study using blood from patients with different disease severity. Proteomics data revealed that IL-6 was higher in critical patients, with proteins of inflammatory pathways and macrophage migration (C5, CHI3L1, LBP, S100A8, S100A9, S100A11, S100A12), neutrophil degranulation (ANXA3, DEFA1B, FGL2, LRG1, PGLYRP1, SLPI), and neutrophil extracellular traps (ELANE, MPO) being gradually augmented with disease severity, as well as LDHA, ADAMTSL4, linking to a positive regulation of apoptosis. Metabolomics analysis highlighted alteration of arginine metabolism with lower levels of arginine, glutamine, and N-acetylornithine across disease severity. Tryptophan metabolism increased in critical patients, with lower levels of tryptophan and higher levels of its metabolites, 5-hydroxyindoleacetic acid, anthranilic acid, kynurenic acid, kynurenine, melatonin, and xanthurenic acid. Finally, the lipidome signature of critical patients showed that ceramides, lysophosphatidylinositol, and PE were higher, while lysoPC were gradually decreased.

The multiomics investigation of Li et al. [78] also highlighted enrichment of immune and infectious pathways, in particular complement activation, inflammatory response, host-virus interaction, and lipid metabolism. Some significant proteins were CETP, CHGA, CCL18, CRP, DEFA1, FGL1, HYOU1, KLKB1, LCAT, MRC1, MBL2, PPBP, RNASE3, S100A8, S100A9, S100A12, SAA1, SPINK5, and TFRC. Combining metabolomics and lipidomics, a reduction in plasma amino acids was observed in SARS-CoV-2-infected groups (alpha-aminobutyric acid, arginine, asparagine, cysteine, glutamic acid, lysine, methionine, serine, threonine, tyrosine, valine), with an increase in lipids as phosphatidylinositols, phosphoserine, DAG, and TG as well as decreased phosphocholine (PC) and phosphoglycerol (PG). A panel of 25 molecules was identified by the authors as predictive of severity, including 4 proteins (CAMP, CLEC3B, GGH, GSN) and 21 lipids, of which 4 are ceramides, 3 glycosylceramides, 4 PC, 4 PE, and 4 TG. 

In addition, the multiomic profiling of the plasma of the 18 COVID-19 children with mild symptoms of Wang et al. [79] detected 44 differential proteins and 249 metabolites, which enriched platelet degranulation, plasminogen activation, fibrinolysis, blood coagulation, extracellular matrix organization as biological processes, and altered biosynthesis of amino acids (arginine metabolism), or other KEGGs such as carbon metabolism, choline metabolism, pyrimidine metabolism, etc. Machine-learning-based inference analysis revealed two combinations of five proteins (ENO1, F9, F11, FGA, FGG) and five metabolites (dihydroorotic acid, indoleacetaldehyde, mannitol, methylmalonic acid, tryptophan) able to accurately discriminate COVID-19 children. ENO1, the only down-regulated amongst the above-mentioned proteins, is a key enzyme of glycolysis required for hypoxia-induced metabolic reprogramming from mitochondrial respiration to glycolysis, while the alteration of the components of coagulation cascade may be associated with the changing levels of other proteins in the dataset (S100A9, SERPINA5, SERPINC1, SERPINF2). Concerning the metabolites, the dihydroorotic acid has involvement in the pyrimidine metabolism and may diminish the injury prompted by glucose metabolism reprogramming upon hypoxia [80]. Methylmalonic acid is a dicarboxylic acid derived from propionate catabolism [81,82], whose role in the regulation of immune and inflammation responses is unclear. Instead, once again tryptophan (and its derivative indoleacetaldehyde) resulted altered in COVID-19.

The serum of COVID-19 patients was extensively characterized by Shen et al. [83] using TMT proteomics and untargeted metabolomics. Whereas severe patients exhibited high levels of CRP and lymphocyte and monocyte suppression, the pathway analysis revealed metabolic and immune alteration, highlighting markers involved in macrophage function (APOA1, APOA2, APOD, APOH, APOL1, APOM), acute phase proteins and complement system (C6, CFB, CFP, CPN1, CRP, ORM1, SAA1, SAA2, SAA4, SAP/APCS, SERPINA3, mannose), and platelet degranulation (PF4, PPBP). The accumulation of 11 steroid hormones that may contribute to disruption of NAD+ synthesis regulation was disclosed by metabolomic analysis. Furthermore, alteration in lipid metabolism was observed, with a decrease in sphingolipids and choline and an increase in PC, as well as an elevation in kynurenic acid, kynurenine, and 8-methoxykynurenate and a decrease in serotonin, confirming alteration of the tryptophan/kynurenine pathway in COVID-19 patients. Amino acid metabolism was profoundly dysregulated, in particular arginine metabolism, which included down-regulation of 2-oxoglutaric acid, arginine, citrulline, fumaric acid, glutamic acid, glutamine, N-(L-arginino)-succinate, N-acetyl-L-glutamic acid, ornithine, and urea.

Cornillet et al. [84] defined a “corrected” metabolome for hospitalized patients by excluding xenobiotics from the analysis of serum, which showed main alterations related to lipid species. Integration of the COVID-19 metabolome with proteomics and other clinical and immunological data highlighted multiple pathways for the immune activation, and organ injury, especially converging to neurological inflammation and tissue damage.

As further confirmation of such alterations, the study from Wilk et al. [85], employing genomics, transcriptomics, and proteomics on the peripheral immune cells collected from a COVID-19 patients’ cohort, disclosed extensive dysregulation of peripheral innate immunity accompanied by a prevalent signature of neutrophil and NK cell hyperactivation, especially in severe and fatal COVID-19.

Finally, considering lung involvement as the main feature of acute damage of SARS-CoV-2 infection, the proteomic and metabolomic analyses of Yang et al. [86] performed on the serum of COVID-19 patients with pulmonary fibrosis disclosed proteins involved in the immune system, cell adhesion, and glycosaminoglycan degradation, and many metabolites involved in the PPAR signaling pathway, D-arginine and D-ornithine metabolism (urea cycle), TRP-inflammatory pathways, and alpha-linolenic acid metabolism. Thus, combined transcriptomics and proteomics analysis identified important genes and proteins, such as the cathepsins CTSB and CTSL associated with SARS-CoV-2 entry and the proinflammatory mediators S100A8, S100A9, S100A11, S100A12, and S100P, which resulted all up-regulated in the fatal cases, enriching neutrophil-related and extracellular structure organization GO terms.

## 5. COVIDomics Data Integration

**Figure 2:**
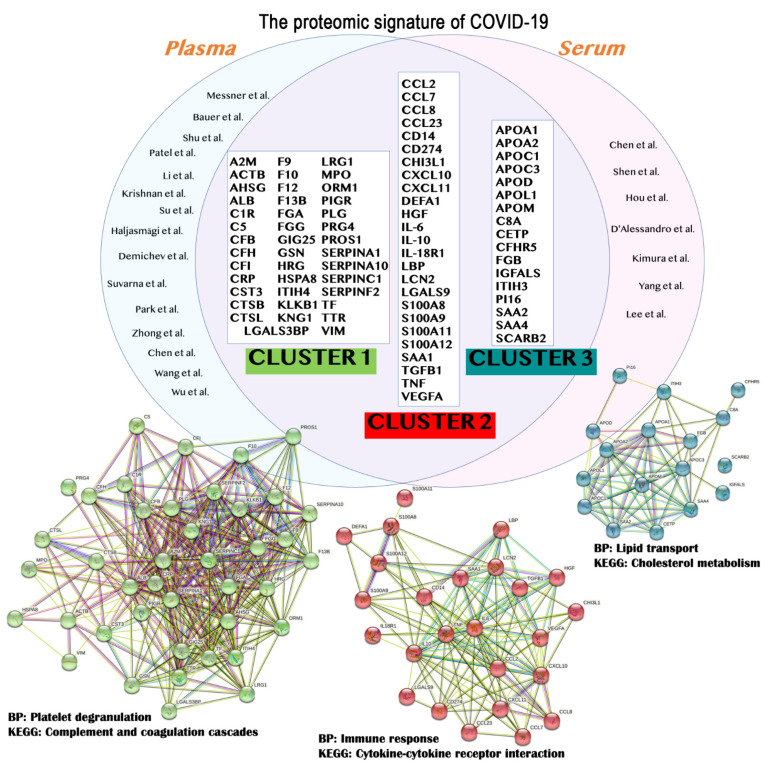
The main findings obtained from the review of proteomics studies are summarized. In particular, the results from plasma [34,35,36,38,39,40,41,42,73,74,75,76,77,78,79] and serum [43,44,45,46,47,83,86] studies were merged to identify the common proteins (top) that should represent the proteome signature of COVID-19. These protein entries were analyzed and clustered using STRING version 11.5, revealing the formation of three main clusters (bottom). The relative biological processes (BP) and Kyoto Encyclopedia of Genes and Genomes (KEGG) pathways were indicated. A detailed list for the proteins depicted in this figure is available in Table 4.

The application of omics and multiomics technologies, enclosed in this review under the term COVIDomics, offers the great chance to meticulously recognize and dissect the multiple molecular aspects that characterize a complex disease such as COVID-19. The majority of patients that contract the SARS-CoV-2 infection may develop no or mild symptoms, although many others experience a severe or critical symptomatology leading to fatal cases. This high heterogeneity in the clinical manifestation is being investigated in many studies, comprising those here reviewed. In fact, many comparisons investigate the differences between the severe disease toward the baseline proteome or metabolome in healthy patients; others focus mostly on the progression of the disease from a condition to another one and highlight correlations of clinical and biochemical parameters with dysregulated proteins and metabolites. Furthermore, the majority of the COVIDomics research was performed on blood, as it is an easily accessible liquid biopsy reflecting the systemic changes subsequent the strong infection of SARS-CoV-2. Thus, the comprehensive and accurate analysis of circulating molecules is helpful to unravel the systemic mechanisms of the disease. In fact, as summarized in Figure 2 and detailed in Table 4, there are several common proteins that were identified in plasma and serum as quantitatively changed between the patients and their controls, representing a relevant proteomic signature of COVID-19. 

To obtain further insights on the biological meaning of the proteome signature of COVID-19, we originally performed functional enrichment of the common protein dataset using STRING software (v. 11.5) [87,88,89]. The bioinformatic analysis produced a full network of protein-protein interactions relevant to COVID-19 for the identification of pathways triggered by the disease. Indeed, in order to assign each protein to a specific biologically meaningful term, the full network was further processed to obtain specific sub-networks. In detail, we used the *kmeans* algorithm as clustering option in STRING software, specifying for the generation of three main clusters as output of the clustering analysis. These clusters, whose network nodes were labelled with cluster-specific colors (Cluster 1: green; Cluster 2: red; Cluster 3: blue), were reported in Figure 2 together with the corresponding proteins. In addition, we searched for each cluster the most relevant Gene Ontology (GO) terms that included the biological processes (BP) and the Kyoto Encyclopedia of Genes and Genomes (KEGG) pathways that are affected in COVID-19 patients. For each cluster, we selected the most significant BP and KEGG, taking into account the following parameters: low *false discovery rate* (FDR), high *strength*, and high *count in network*. Accordingly, the Cluster 1 enriched *Platelet degranulation* as main BP (FDR = 3.78 × 10^–20^) and *Complement and coagulation cascades* as main KEGG (FDR = 5.66 × 10^–31^). The Cluster 2 highlighted *Immune response* as principal BP (FDR = 7.02 × 10^–18^) and *Cytokine-cytokine receptor interaction* as significant KEGG (FDR = 1.88 × 10^–10^). Finally, the BP *Lipid transport* (FDR = 3.66 × 10^–9^) and the KEGG *Cholesterol metabolism* (FDR = 3.28 × 10^–7^) were significantly enriched for Cluster 3. Our findings bring up the general biochemical dysregulations that take place in the blood of COVID-19 patients as reflection of the systemic perturbation caused by SARS-CoV-2 infection. Accordingly, the alterations in the immune function, coagulation system, and lipid metabolic processes have been extensively reviewed by the recent literature [90,91,92,93,94,95,96].

**Figure 3:**
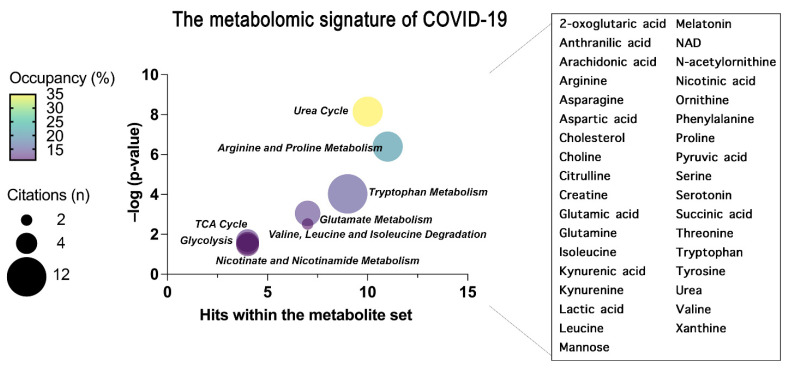
The common metabolites and metabolic pathways obtained from the review of plasma and serum metabolomics studies are summarized. A specific signature of the metabolome of COVID-19 patients was obtained representing the most enriched pathways constructed through an over-representation analysis (ORA) within MetaboAnalyst 5.0 and represented as bubble plot. The bubble plot takes into account the statistical significance (–log *p*-value) of each metabolite set identified through the ORA analysis. Where the size of each bubble refers to the number of times the metabolite set is cited in this review by each author, the color instead refers to the number of metabolites detected over the total number of metabolites within that pathway (occupancy). On the right, the metabolites common to all the reviewed papers that have generated the pathway enrichment are grouped.

As for proteins, we have also summarized the common findings obtained reviewing metabolomics studies in terms of metabolites and metabolic pathways highlighted in plasma and serum from COVID-19 patients (Figure 3). In particular, we have focused our analysis on small molecules, since metabolomics software only recognize well-annotated HMDB (Human Metabolome Database) compounds, and lipids may be not correctly mapped. Indeed, the enrichment analysis of the metabolites associated with COVID-19 was performed using MetaboAnalyst 5.0 software [97,98,99,100].

In detail, a Metabolite Set Enrichment Analysis (MSEA) was carried out to highlight the biological meaning of the selected metabolomics data. To achieve this goal, an Over Representation Analysis (ORA) algorithm was used selecting KEGG-based pathways as metabolite set library and using metabolite sets containing at least 2 entries. 

This analysis, reported as bubble plot in Figure 3, highlighted the metabolites shared by all the reviewed studies and revealed that several metabolic dysregulations seem to affect COVID-19 patients. The significant KEGG pathways retrieved from ORA analysis were selected including significant (–log *p*-value > 1.3) and non-redundant terms. The identified metabolic dysregulations involve several amino acid metabolic pathways, including arginine and proline metabolism, glutamate metabolism, and tryptophan metabolism. Moreover, defects in energy metabolism, including carbon and nitrogen metabolism (TCA cycle, urea cycle, glycolysis) appear to be prevalently altered in infected individuals [101]. Details of the metabolite sets and relative KEGG pathways are listed in Table 5. Even if not resumed, also lipid metabolism is extensively dysregulated in COVID-19 patients [102,103].

## 6. Concluding Remarks

During the current COVID-19 pandemic, unprecedented efforts have been made by the scientific community to dissect the molecular bases of SARS-CoV-2 infection, spreading, and pathogenicity. Omics scientists deserved particular merits for performing several proteomics- and metabolomics-based investigations, but also integrative multiomics analyses, using samples derived from patients. Thus, the application of COVIDomics strategies has certainly empowered the knowledge relative to COVID-19 in terms of biomarkers of disease and specific physiological mechanisms disturbed upon SARS-CoV-2 infection. The study of coronavirus structure, replication, and infection, together with the virus-host interaction and the host response, have remarkably provided us with efficient tools to diagnose the disease and ameliorate the fate of COVID-19 patients. In general, a huge improvement in patients’ outcomes is correlated to the development of vaccines as prophylactic therapeutic approaches, despite it remaining not currently possible to foresee the prognosis of infected patients by a single analysis or through the detection of a single and specific marker. Nonetheless, in this scenario, the use of omics sciences and their integration have provided us with relevant proteomics and metabolomics signatures, as a point of junction between SARS-CoV-2 infection/pathobiology with the subsequent clinical outcome of affected patients. Thus, COVIDomics is contributing to a better comprehension of the molecular intricacy of COVID-19, in the view of identifying new molecular actors involved in the pathogenesis of the disease to be used for efficient therapeutic approaches in combatting the COVID-19 pandemic.

## Figures and Tables

**Table 1:** Summary of the main characteristics of the proteomics publications related to COVID-19 patients.

Authors	Biologic Matrix	Patients	Technology	Pathway/Protein Dysregulation
Bauer et al. (2021)	Plasma	44 non-hospitalized COVID-19 53 hospitalized COVID-19 44 non-COVID-19	PEA	Inflammation
Chen Y. et al. (2021)	Serum	10 moderate COVID-19 6 severe COVID-19 10 healthy controls	DIA-MS	Cholesterol metabolism, coagulation, cardiovascular system
D’Alessandro et al. (2020)	Serum	33 COVID-19 16 non-COVID-19	LC-MS/MS	IL-6 signaling, coagulation, complement, antimicrobial enzymes
Demichev et al. (2021)	Plasma	139 (WHO grade 3–7) COVID-19 16 non-COVID-19	DIA-MS	Coagulation, complement, immune system, inflammation
Haljasmägi et al. (2020)	Plasma	25 moderate COVID-19 15 severe COVID-19 18 healthy controls	PEA	Apoptosis, inflammation, neuronal injury
Hou et al. (2020)	Serum	15 COVID-19 13 influenza	antibody microarray	Immune system, inflammation
Kimura et al. (2021)	Serum	10 severe COVID-19	DIA-MS	cardiovascular system, inflammation
Lee et al. (2021)	Serum	13 non-severe COVID-19 12 severe COVID-19	DIA-MS	Coagulation, immune system, inflammation, lipid metabolism
Messner et al. (2021)	Plasma	31 (mild + severe) COVID-19 + 17 COVID-19 15 healthy controls	DIA-MS	Coagulation, complement, inflammation
Park et al. (2020)	Plasma	3 mild COVID-19 5 severe COVID-19	LC-MS/MS	Coagulation, neutrophils activation
Patel et al. (2021)	Plasma	26 mild COVID-19 9 severe COVID-19 24 critical COVID-19 28 healthy controls	PEA	Cytokine-cytokine receptor interaction
Shu et al. (2020)	Plasma	10 mild COVID-19 7 severe COVID-19 5 fatal COVID-19 8 healthy controls	LC-MS/MS	Coagulation, complement, energy metabolism, immune system, inflammation
Zhong et al. (2021)	Plasma	50 (mild + moderate) COVID-19	PEA	Cytokine-related, immune-related

The papers are ordered alphabetically by author name. DIA = data-independent acquisition; LC-MS/MS = liquid chromatography—tandem mass spectrometry; PEA = proximity extension assay; WHO = World Health Organization.

**Table 2:** Summary of the main characteristics of the metabolomics and lipidomics publications related to COVID-19 patients.

Authors	Biologic Matrix	Patients	Technology	Pathway/Metabolite Dysregulation
Ansone et al. (2021)	Serum	32 hospitalized COVID-19 39 healthy controls	LC-MS/MS	Amino acid metabolism, tryptophan metabolism, urea cycle
Bizkarguenaga et al. (2021)	Plasma	69 recovered COVID-19 71 healthy controls	NMR	TG, cholesterol, phospholipids
Blasco et al. (2020)	Plasma	55 COVID-19 45 healthy controls	LC-MS/MS	NAD metabolism, pyrimidine metabolism, tryptophan metabolism
Caterino M. et al. (2021)	Serum	20 mild COVID-19 16 moderate COVID-19 17 severe COVID-19 9 healthy controls	LC-MS/MS	Carbon and nitrogen metabolism, energy metabolism, purine and pyrimidine metabolism
Caterino M. et al. (2021)	Serum	20 mild COVID-19 16 moderate COVID-19 16 severe COVID-19 9 healthy controls	LC-MS/MS	Cer, TG
Danlos et al. (2021)	Plasma	23 mild COVID-19 21 moderate COVID-19 28 critical COVID-19 29 healthy controls	GC-MS LC-MS/MS	Tryptophan metabolism
Dei Cas et al. (2021)	Serum	49 COVID-19 10 healthy controls	LC-MS/MS	Acylcarnitines, PC, PE, CE, DAG, lysoPE, SM
Fraser et al. (2020)	Plasma	10 COVID19+ patients 10 COVID19– patients 10 healthy controls	LC-MS/MS NMR	Tryptophan metabolism, lysoPC
Jia et al. (2021)	Serum	18 mild COVID-19 12 severe COVID-19 69 recovered COVID-19 13 healthy controls + 90 COVID-19 28 healthy controls	LC-MS/MS	Amino acid metabolism, TCA cycle, urea cycle
Kaur et al. (2021)	Serum	6 COVID-19 6 recovered COVID-19	LC-MS/MS	PC, SM, arachidonic acid, tryptophan metabolism
Khodadoust et al. (2021)	Plasma	32 mild COVID-19 18 severe COVID-19 (n.f.) healthy controls	LC-MS/MS	Cer
Li T. et al. (2021)	Serum	30 (mild + moderate) COVID-19 17 severe COVID-19 20 healthy controls	LC-MS/MS	Amino acid metabolism, carbohydrate metabolism, urea cycle
Páez-Franco et al. (2021)	Serum	19 mild COVID-19 46 severe COVID-19 27 healthy controls	GC-MS	Amino acid metabolism, energy metabolism
Roberts et al. (2021)	Serum	71 mild COVID-19 49 severe COVID-19 + 90 COVID-19	LC-MS/MS	Acylcarnitines, energy metabolism, tryptophan metabolism
Shi et al. (2021)	Serum	79 COVID-19 30 COVID-19-like 78 healthy controls	GC-MS	Amino acid metabolism, energy metabolism
Sindelar et al. (2021)	Plasma	272 COVID-19 67 negative controls	LC-MS/MS	Cer, lysoPC, PC
Thomas et al. (2020)	Serum	33 COVID-19 16 negative controls	LC-MS/MS	Carbon and nitrogen metabolism, tryptophan metabolism
Torretta et al. (2021)	Serum	11 mild COVID-19 28 moderate COVID-19 12 severe COVID-19 8 critical COVID-19 27 healthy controls	LC-MS/MS	Cer, SM, sphingosine
Xiao et al. (2021)	Serum	14 mild COVID-19 (+7 mild COVID-19) 23 severe COVID-19 17 healthy controls	LC-MS/MS	Arginine metabolism, purine metabolism, tryptophan metabolism

The reviewed papers are ordered alphabetically by author name. CE = cholesteryl esters; Cer = ceramides; DAG = diacylglycerols; GC-MS = gas chromatography-mass spectrometry; LC-MS/MS = liquid chromatography—tandem mass spectrometry; lysoPC = lysophosphatidylcholines; lysoPE = phosphatidylethanolamines; n.f. = not found; NMR = nuclear magnetic resonance; PC = phosphatidylcholines; PE = phosphatidylethanolamines; SM = sphingomyelins; TCA = tricarboxylic acids; TG = triglycerides.

**Table 3:** Summary of the main characteristics of the multiomics publications containing proteomics and/or metabolomics studies related to COVID-19 patients.

Authors	Biologic Matrix	Patients	Omics Used	Technology	Proteomic/Metabolomic Dysregulation
Chen Y.-M. et al. (2020)	Plasma	50 mild COVID-19 16 severe COVID-19 17 healthy controls	Proteomics Metabolomics	DIA-MS NMR	TCA cycle, glycolytic pathway, platelet signaling pathway, TG, cholesterol, phospholipids
Cornillet et al. (2021)	Serum	27 (moderate + severe) COVID-19 17 healthy controls	Proteomics Metabolomics	PEA LC-MS/MS	Immune system, neurological inflammation
Krishnan et al. (2021)	Plasma	41 (mild + severe) COVID-19 31 healthy controls	Proteomics Metabolomics	PEA LC-MS/MS	Cytokine-cytokine receptor interaction, chemokine signaling, TNF signaling pathway, glycolysis, TCA cycle
Li Y. et al. (2021)	Plasma	10 non-severe COVID-19 10 severe COVID-19 10 healthy controls + 5 non-severe COVID-19 5 severe COVID-19	Proteomics Metabolomics	DIA-MS LC-MS/MS	Complement, inflammation, host-virus interaction, lipid metabolism, DAG, TG, PC, PG
Shen et al. (2020)	Serum	25 non-severe COVID-19 28 severe COVID-19 25 non-COVID-19 25 healthy controls	Proteomics Metabolomics	LC-MS/MS LC-MS/MS	Coagulation, complement, immune system, inflammation, arginine metabolism, lipid metabolism, NAD and tryptophan metabolism
Su et al. (2020)	Plasma	139 COVID-19 133 healthy controls	Proteomics Metabolomics	PEA LC-MS/MS	Amino acid metabolism, tryptophan metabolism, urea cycle
Suvarna et al. (2021)	Plasma	13 COVID-19	Proteomics Metabolomics	LC-MS/MS LC-MS/MS	Coagulation, complement, myeloid leukocyte activation, arginine amino acid metabolism
Wang et al. (2021)	Plasma	18 mild COVID-19 12 healthy controls	Proteomics Metabolomics	LC-MS/MS LC-MS/MS	Coagulation, extra-cellular matrix organization, arginine metabolism, carbon metabolism, choline metabolism, pyrimidine and tryptophan metabolism
Wilk et al. (2021)	Blood (immune cells)	64 (mild-to-fatal) COVID-19 8 healthy controls	Proteomics	CyTOF	Immune system, neutrophil and NK cell hyperactivation
Wu et al. (2021)	Plasma	231 (asymptomatic, mild, severe, critical) COVID-19	Proteomics Metabolomics	DIA-MS LC-MS/MS	Inflammation, macrophage migration, neutrophil degranulation, apoptosis, arginine metabolism, tryptophan metabolism, Cer, lysoPC, PE
Yang et al. (2021)	Serum	85 COVID-19 41 non-pulmonary fibrosis 44 pulmonary fibrosis	Proteomics Metabolomics	DIA-MS LC-MS/MS	Immune system, cell adhesion, PPAR signaling, D-arginine and D-ornithine metabolism (urea cycle)

The papers were ordered alphabetically by author name. CyTOF = cytometry by time of flight; Cer = ceramides; DAG = diacylglycerols; DIA = data-independent acquisition; LC-MS/MS = liquid chromatography—tandem mass spectrometry; lysoPC = lysophosphatidylcholines; NMR = nuclear magnetic resonance; PC = phosphatidylcholines; PE = phosphatidylethanolamines; PEA = proximity extension assay; PG = phosphoglycerols; TCA = tricarboxylic acids; TG = triglycerides.

**Table 4:** The list of the proteins identified in more than one study characterizing the blood proteomic signature of COVID-19.

Protein Symbol	UniProt ID	Protein Name	STRING Cluster
A2M	P01023	Alpha-2-macroglobulin	Cluster 1
ACTB	P60709	Actin, cytoplasmic 1	
AHSG	P02765	Alpha-2-HS-glycoprotein	
ALB	P02768	Albumin	
C1R	P00736	Complement C1r subcomponent	
C5	P01031	Complement C5	
CFB	P00751	Complement Factor B	
CFH	P08603	Complement factor H	
CFI	P05156	Complement factor I	
CRP	P02741	C-reactive protein	
CST3	P01034	Cystatin-C	
CTSB	P07858	Cathepsin B	
CTSL	P07711	Procathepsin L	
F9	P00740	Coagulation factor IX	
F10	P00742	Coagulation factor X	
F12	P00748	Coagulation factor XII	
F13B	P05160	Coagulation factor XIII B chain	
FGA	P02671	Fibrinogen alpha chain	
FGG	P02679	Fibrinogen gamma chain	
GSN	P06396	Gelsolin	
HRG	P04196	Histidine-rich glycoprotein	
HSPA8	P11142	Heat shock cognate 71 kDa protein	
ITIH4	Q14624	Inter-alpha-trypsin inhibitor heavy chain H4	
KLKB1	P03952	Plasma kallikrein	
KNG1	P01042	Kininogen-1	
LGALS3BP	Q08380	Galectin-3-binding protein	
LRG1	P02750	Leucine-rich alpha-2-glycoprotein	
MPO	P05164	Myeloperoxidase	
ORM1	P02763	Alpha-1-acid glycoprotein 1	
PIGR	P01833	Polymeric immunoglobulin receptor	
PLG	P00747	Plasminogen	
PRG4	Q92954	Proteoglycan 4	
PROS1	P07225	Vitamin K-dependent protein S	
SERPINA1	P01009	Alpha-1-antitrypsin	
SERPINA3	P01011	Alpha-1-antichymotrypsin	
SERPINA10	Q9UK55	Protein Z-dependent protease inhibitor	
SERPINC1	P01008	Antithrombin-III	
SERPINF2	P08697	Alpha-2-antiplasmin	
TF	P02787	Transferrin	
TTR	P02766	Transthyretin	
VIM	P08670	Vimentin	
CCL2	P13500	C-C motif chemokine 2	Cluster 2
CCL7	P80098	C-C motif chemokine 7	
CCL8	P80075	C-C motif chemokine 8	
CD14	P08571	Monocyte differentiation antigen CD14	
CCL23	P55773	C-C motif chemokine 23	
CD274	Q9NZQ7	Programmed cell death 1 ligand 1	
CHI3L1	P36222	Chitinase-3-like protein 1	
CXCL10	P02778	C-X-C motif chemokine 10	
CXCL11	O14625	C-X-C motif chemokine 11	
DEFA1	P59665	Neutrophil defensin 1	
HGF	P14210	Hepatocyte growth factor	
IL-10	P22301	Interleukin-10	
IL-18R1	Q13478	Interleukin-18 receptor 1	
IL-6	P08887	Interleukin-6 receptor subunit alpha	
LBP	P18428	Lipopolysaccharide-binding protein	
LCN2	P80188	Neutrophil gelatinase-associated lipocalin	
LGALS9	O00182	Galectin-9	
S100A11	P31949	Protein S100-A11	
S100A12	P80511	Protein S100-A12	
S100A8	P05109	Protein S100-A8	
S100A9	P06702	Protein S100-A9	
SAA1	P0DJI8	Serum amyloid A-1 protein	
TGFB1	P01137	Transforming growth factor beta-1 proprotein	
TNF	P01375	Tumor necrosis factor	
VEGFA	P15692	Vascular endothelial growth factor A	
APOA1	P02647	Apolipoprotein A1	Cluster 3
APOA2	P02652	Apolipoprotein A2	
APOC1	P02654	Apolipoprotein C1	
APOC3	P02656	Apolipoprotein C3	
APOD	P05090	Apolipoprotein D	
APOL1	O14791	Apolipoprotein L1	
APOM	O95445	Apolipoprotein M	
C8A	P07357	Complement component C8 alpha chain	
CETP	P11597	Cholesteryl ester transfer protein	
CFHR5	Q9BXR6	Complement factor H-related protein 5	
FGB	P02675	Fibrinogen beta chain	
IGFALS	P35858	Insulin-like growth factor-binding protein complex acid labile subunit	
ITIH3	Q06033	Inter-alpha-trypsin inhibitor heavy chain H3	
PI16	Q6UXB8	Peptidase inhibitor 16	
SAA2	P0DJI9	Serum amyloid A-2 protein	
SAA4	P35542	Serum amyloid A-4 protein	
SCARB2	Q14108	Lysosome membrane protein 2	

Proteins were ordered alphabetically for each cluster according to the protein symbol.

**Table 5:** Details of the metabolite sets enriching the KEGG pathways that constitute the metabolic signature of COVID-19.

KEGG Pathway	Metabolite Set
Urea Cycle	2-oxoglutaric acid, Arginine, Aspartic acid, Citrulline, Glutamic acid, Glutamine, NAD, Ornithine, Pyruvic acid, Urea
Arginine and Proline Metabolism	2-oxoglutaric acid, Arginine, Aspartic acid, Citrulline, Glutamic acid, NAD, Ornithine, Proline, Succinic acid, Urea
Tryptophan Metabolism	2-oxoglutaric acid, Anthranilic acid, Glutamic acid, Kynurenic acid, Kynurenine, Melatonin, NAD, Serotonin, Tryptophan
Glutamate Metabolism	2-oxoglutaric acid, Aspartic acid, Glutamic acid, Glutamine, NAD, Pyruvic acid, Succinic acid
Valine, Leucine and Isoleucine Degradation	2-oxoglutaric acid, Glutamic acid, Isoleucine, Leucine, NAD, Succinic acid, Valine
TCA Cycle	2-oxoglutaric acid, NAD, Pyruvic acid, Succinic acid
Glycolysis	2-oxoglutaric acid, Lactic acid, NAD, Pyruvic acid
Nicotinate and Nicotinamide Metabolism	Glutamic acid, Glutamine, NAD, Nicotinic acid

KEGG pathways were ordered according to decrescent statistical significance.

## Data Availability

Not applicable.
